# Performance impact of mutation operators of a subpopulation-based genetic algorithm for multi-robot task allocation problems

**DOI:** 10.1186/s40064-016-3027-2

**Published:** 2016-08-18

**Authors:** Chun Liu, Andreas Kroll

**Affiliations:** 1 School of Automation, Beijing University of Posts and Telecommunications, No 10, Xitucheng Road, 100876 Beijing, China; 2 Department of Measurement and Control, Mechanical Engineering, University of Kassel, Mönchebergstraße 7, 34125 Kassel, Germany

**Keywords:** Multi-robot task allocation, Genetic algorithms, Constrained combinatorial optimization, Mutation operators, Subpopulation

## Abstract

Multi-robot task allocation determines the task sequence and distribution for a group of robots in multi-robot systems, which is one of constrained combinatorial optimization problems and more complex in case of cooperative tasks because they introduce additional spatial and temporal constraints. To solve multi-robot task allocation problems with cooperative tasks efficiently, a subpopulation-based genetic algorithm, a crossover-free genetic algorithm employing mutation operators and elitism selection in each subpopulation, is developed in this paper. Moreover, the impact of mutation operators (swap, insertion, inversion, displacement, and their various combinations) is analyzed when solving several industrial plant inspection problems. The experimental results show that: (1) the proposed genetic algorithm can obtain better solutions than the tested binary tournament genetic algorithm with partially mapped crossover; (2) inversion mutation performs better than other tested mutation operators when solving problems without cooperative tasks, and the swap-inversion combination performs better than other tested mutation operators/combinations when solving problems with cooperative tasks. As it is difficult to produce all desired effects with a single mutation operator, using multiple mutation operators (including both inversion and swap) is suggested when solving similar combinatorial optimization problems.

**Electronic supplementary material:**

The online version of this article (doi:10.1186/s40064-016-3027-2) contains supplementary material, which is available to authorized users.

## Introduction

Multi-robot task allocation (MRTA) determines the task distribution and schedule for a group of robots in multi-robot systems (Gerkey and Matarić 2004). It is a constrained combinatorial optimization problem, which usually provides solutions to minimize the cost or maximize the profit while satisfying some operational constraints. MRTA problems without cooperative tasks are similar to multiple traveling salesman problems, both of them are NP- (non-deterministic polynomial-time) hard optimization problems. MRTA problems with cooperative tasks are more complex and strongly NP-hard (Gerkey and Matarić 2004), because each cooperative task requires at least two robots to carry it out simultaneously, which introduces both spatial and temporal constraints.

For solving an MRTA problem, the first important thing is to understand what the tasks are. In general, tasks in an MRTA problem can be classified into single-robot tasks and multi-robot tasks (Gerkey and Matarić 2004). A single-robot task is carried out by a single robot. A multi-robot task requires multiple robots to perform, which is also referred to as cooperative task. Tasks vary in different practical applications. Moreover, the problem complexity increases with the number of robots required for each cooperative task.

In the industrial plant inspection, two types of the inspection tasks exist according to the measurement method and detection sensors (Bonow and Kroll 2013; Ordoñez Müller and Kroll 2013): single-robot tasks and two-robot tasks (cooperative tasks, each of which requires two robots to carry out simultaneously). This paper aims at developing an efficient algorithm to solve the multi-robot tasks allocation problems for the industrial plant inspection.

The contributions of this paper include: (1) the development of the subpopulation-based genetic algorithm, this algorithm employs mutation operators and elitism selection in each subpopulation, and can solve the multi-robot tasks allocation problems for industrial plant inspection efficiently; and (2) the recommendation of mutation operators for solving multi-robot task allocation problems and similar optimization problems, i.e., using multiple mutation operators (including both inversion and swap) is suggested.

## Background

### Related works on multi-robot task allocation problems

To find good solutions efficiently for multi-robot task allocation problems, many approaches have been developed, such as genetic algorithms (Jones et al. 2011; Liu and Kroll 2012a), hybrid genetic algorithms (Liu and Kroll 2015; Ni and Yang 2012), auction-based algorithms (Das et al. 2015), behavior-based algorithms (Butler and Hays 2015), negotiations-based approaches (Rossi et al. 2015). Some of them can solve MRTA problems both without and with cooperative tasks, but some only can solve problems without cooperative tasks.

Goldberg et al. (2003) proposed a distributed layered architecture for multi-robot systems. This architecture includes three layers: planning layer, executive layer, and behavior layer. In the planning layer, a market-based approach was developed to allocate tasks. Based on the market economy, Dias (2004), Zhang and Parker (2013) developed TraderBots and IQ-ASyMTRe. Cicirello and Smith (2001, 2002) presented a decentralized mechanism for coordinating factory operations in behavioral models. Similar behavior-based algorithms include ALLIANCE (Parker 1998) and BLE (Werger and Matarić 2000). These methods can quickly deal with new tasks and dynamic environmental information during execution. Different from distributed/decentralized methods mentioned above, this paper focuses on centralized approaches and aims at providing the optimal solution for multi-robot task allocations with cooperative tasks.

### Related works on genetic algorithms

A genetic algorithm (GA) (Mitchell 1998) is a centralized heuristic method inspired by biological evolution. It is widely used for optimization and search problems because of its simplicity, high flexibility in problem modeling, and good global search capability. Many genetic algorithms have been developed to solve optimization problems in computational science, engineering, economics, and other fields. For example, in engineering applications, genetic algorithms have been used to solve the design of roof structures (Kociecki and Adeli 2014), assembly problems (Akpınar and Bayhan 2011), and industrial plant inspection planning problems (Liu and Kroll 2012a).

Selection, crossover, and mutation operators maintain the population diversity (Mc Ginley et al. 2011), and also influence the performance of genetic algorithms. Therefore, many efforts have been devoted to the design of these operators; for example, a new selection strategy based on population recombination and elitist refinement (Kwak and Lee 2011), a two-part chromosome crossover operator (Yuan et al. 2013), and a greedy sub tour mutation operator (Albayrak and Allahverdi 2011) have been developed to improve the efficiency of genetic algorithms. Crossover and mutation are the main search operators of genetic algorithms. They play different roles in genetic algorithms: crossover tends to preserve the features of the parents, while mutation tends to make some small local perturbation of individuals. Compared to crossover, mutation is usually considered as a secondary operator with a low probability in standard genetic algorithms (Holland 1992). This could be due to the fact that a large mutation rate would make genetic algorithms to search randomly. However, many studies have shown that genetic algorithms without crossover can perform better than standard genetic algorithms, if mutation is combined with an effective selection operator (Fogel and Atmar 1990; Liu and Kroll 2012b; Osaba et al. 2014; Walkenhorst and Bertram 2011).

Mutation is carried out with a single parent and plays an important role in increasing the population diversity. Various mutation operators have been developed for different solution representations: bit inversion mutation for binary coding (Holland 1992); swap, insertion, inversion and displacement for permutation coding (Larrañaga et al. 1999); Gaussian mutation (Sarangi et al. 2015), polynomial and power mutation for real coding (Deb and Deb 2012; Deep and Thakur 2007). Some mutation operators are problem-dependent, such as greedy sub tour mutation for traveling salesman problems (Albayrak and Allahverdi 2011) and energy mutation for multicast routing problems (Karthikeyan et al. 2013). Some studies suggest a mutation-combination (Deep and Mebrahtu 2011) or self-adaptive mutation operators (Hong et al. 2000; Mc Ginley et al. 2011; Serpell and Smith 2010). The performance of different mutation operators highly depends on the parameter choice of genetic algorithms (Brizuela and Aceves 2003; Osaba et al. 2014; Wang and Zhang 2006) and the type of problems (Hasan and Saleh 2011; Karthikeyan et al. 2013). Most of related work studied problems without cooperative tasks, such as traveling salesman problems (Albayrak and Allahverdi 2011; Deep and Mebrahtu 2011) and flow shop scheduling (Nearchou 2004; Wang and Zhang 2006). In this paper, the performance of mutation operators will be analyzed when solving multi-robot task allocation problems without or with cooperative tasks.

Permutation coding is used to represent solutions in this paper, which will be illustrated in section “Methods”. As a natural coding, permutation representation is widely used for many search and optimization problems, such as traveling salesman problems, vehicle routing problems, job scheduling problems, task assignment problems. Permutation problems can be classified into three types according to what influences solution fitness (Cicirello 2015, 2016; Cicirello and Cernera 2013; Hernando et al. 2016; Schiavinotto and Stützle 2007; Sörensen 2007; Tayarani-N. and Prügel-Bennett 2014): A-permutation (absolute element positions most impact fitness), R-permutation (relative ordering most impacts fitness), and P-permutation (elements’ precedence impacts fitness). Cicirello (2016) theoretically analyzed the performance of several common mutation operators on different permutation problems. Multi-robot task allocation problems studied in this paper are blended-permutation problems as both task assignments among robots and task scheduling for each robot impact the fitness. The performance of different mutation operators on solving these problems will also be analyzed in this paper.

## Multi-robot task allocation problems for industrial plant inspection

### Characteristics of tasks

**Fig. 1:**
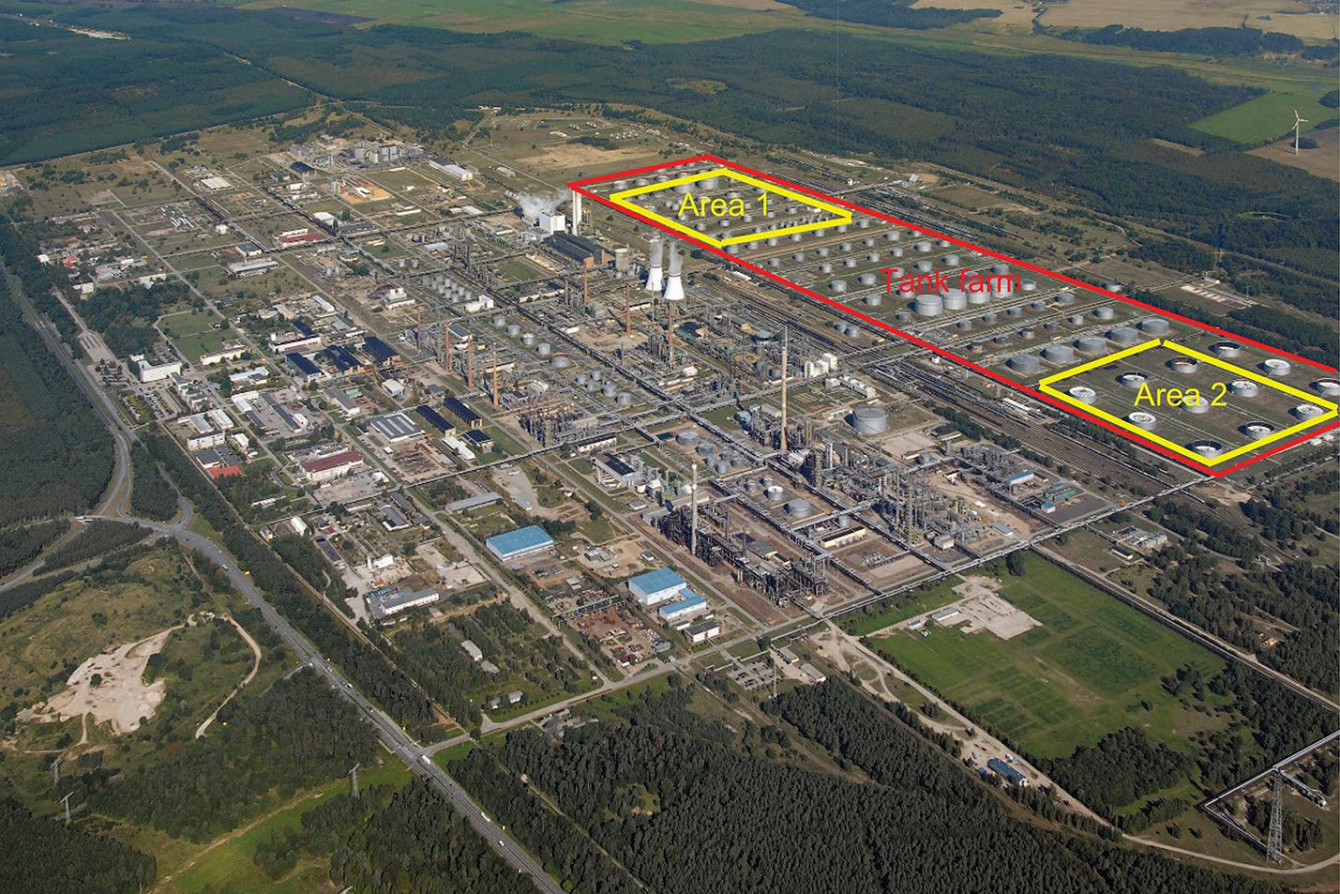
PCK refinery (© PCK Raffinerie GmbH)

This paper studies the problem of multi-robot task allocation for industrial plant inspection, and the target scenarios are derived from a tank farm of a petroleum refinery (Fig. 1). To detect gas and fluid leakages in this refinery, remote sensing is usually used (Bonow and Kroll 2013; Ordoñez Müller and Kroll 2013). This applied sensing technology requires an active sensor and a diffuse reflecting background, and they are located in different positions within their measurement range. If the surface of a target object can be used as a reflecting background, only one robot with an active sensor is required to detect the leakage of this target object; otherwise, two robots (one robot with an active sensor and the other assistant robot with a special retro-reflector (Ordoñez Müller and Kroll 2014) are required. This results in two types of tasks: single- and two-robot tasks. Each single-robot task is performed by one robot with an active sensor at an inspection position. Each two-robot task is carried out by two distinct robots at two inspection positions simultaneously. Multi-robot task allocation for inspection problems with cooperative tasks (two-robot tasks) introduces spatial and temporal constraints: *spatial constraints*, tasks must be executed by robots each from specific inspection position; *temporal constraints*, each cooperative task requires two robots to carry out at the very same time.

**Fig. 2:**
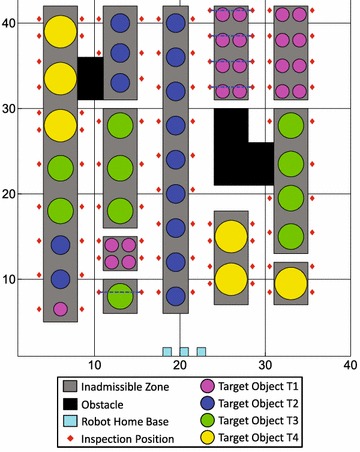
Inspection area and tasks of Prob.C. Two inspection position of each cooperative task linked by a *dashed line*

**Fig. 3:**
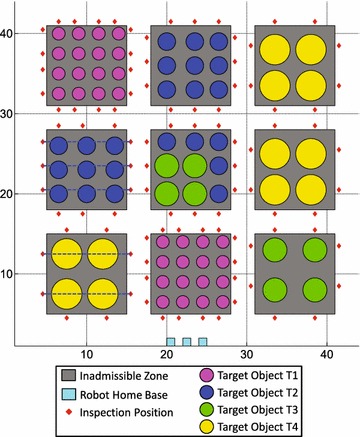
Inspection area and tasks of Prob.D. Two subtasks of each cooperative task linked by a *dashed line*

Figures 2 and 3 display the simulation environments of two inspection problems with cooperative tasks, which are derived from “Area 1” and “Area 2” of Fig. 1, respectively. Both inspection problems are accomplished by 3 mobile robots. The inspection time of the robots at inspection positions (marked with diamonds) is determined by the diameters of target objects. All robots can move freely outside the inadmissible zones and obstacles (marked with rectangles). Each pair of diamonds linked by a bold dashed line defines the two inspection positions of a two-robot task (cooperative task). The other diamonds represent inspection positions of single-robot tasks.

### Assumptions of the problem

This paper focuses on strategic planning that makes decisions on allocating tasks to robots, and does not respond to unplanned events such as robot collisions or road blocks that can be handled during tactical planning. The multi-robot task allocation problems studied in this paper are performed based on the following assumptions. (1) All robots are identical and start at the same time. (2) All robots start from their home bases that are predefined, and end at their home bases after finishing their assigned tasks. Between performing any two tasks, robots do not move back to their home bases. (3) Each single-robot task is assigned to one robot, while each cooperative task is assigned to two robots. Based on the described characteristics and assumptions of the multi-robot task allocation for inspection problems, the time of each robot finishing its assigned tasks includes three parts: the traveling time, the inspection time of each task, and the waiting time occurring when performing cooperative tasks. In this work, the traveling time is calculated using a modified A* algorithm (Liu 2014) that corrects infeasible paths caused by the standard A* algorithm (Hart et al. 1968). The inspection time is predefined according to the inspection method and measurement system properties. The waiting time depends on the solution itself and is calculated for each solution candidate during the execution of the genetic algorithm.

### Objective and constraints

The objective of multi-robot task allocation problems is usually to minimize the total mission cost due to energy consumption, completion time, and/or traveled distance. Shorter duration of inspecting a plant permits a higher frequency of repetition of the inspections, and also may reduce costs of the inspection. Therefore, the objective of multi-robot task allocation problems in this paper is to find the optimal solution $\mathbf A _{opt}$ that requires the minimal completion time (the time span between the first robot starting its work and the last robot finishing its tasks). $\mathbf A _{opt} \in \mathbb {A}$, $\mathbb {A}$ is the set of all admissible solutions of the task allocation problem.

**Fig. 4:**
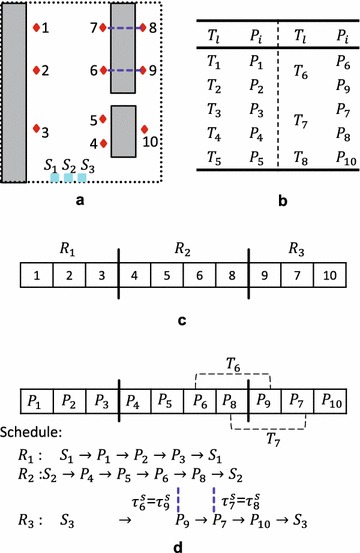
An example with single-robot tasks ($T_1-T_5,T_8$) and cooperative tasks ($T_6,T_7$). **a** Example map. **b** Tasks and subtasks. **c** Genotype. **d** Phenotype

Formally, given a set of $N^R$ identical robots $R=\{R_k \vert k\in \{1,2,\ldots ,N^R\}\}$ and a set of $N^T$ tasks $T=\{T_l \vert l\in \{1,2,\ldots ,N^T\}\}$, the completion time (fitness value) *J* of each admissible solution $\mathbf A \in \mathbb {A}$ can be represented as

1 $$\begin{aligned} J(\mathbf A )=\mathop {\max }\limits _{k\in \{1,\ldots ,N^R\}} C_k (A_k ), \end{aligned}$$

where $\mathbf A =\{A_k \vert k\in \{1,2,\ldots ,N^R\}\}$ denoted as a set of $N^R$ vectors, $A_k$ is the task sequence of robot $R_k$ and denoted as a vector, and $C_k (A_k )$ is the time of robot $R_k$ required to finish all assigned tasks according to the sequence $A_k$. As we assume that all robots start at the same time, the completion time is the maximal/longest value of operation times of all robots in the system.

**Fig. 5:**
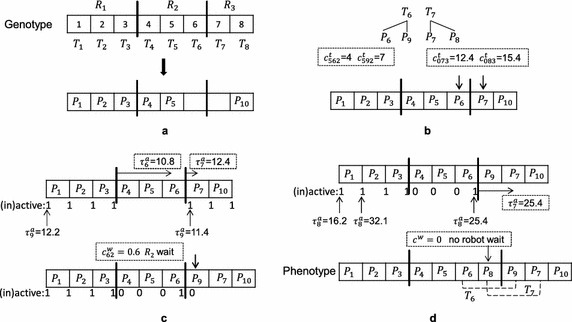
Decoding for a genotype in Fig. 4. **a** Decode single-robot tasks. **b** Decode a subtask of each cooperative task. **c** Decode the other subtask *P*_9_ of the first cooperative task *T*_6_. **d** Decode the other subtask *P*_8_ of the second cooperative task *T*_7_

For task allocation problems with only single-robot tasks, instead of $\mathbf A$ in Eq. (1), each admissible solution is denoted as $\mathbf A ^S = \{A_k^S \vert k\in \{1,2,\ldots ,N^R\}\}$ that has to satisfy the following constraint:

2 $$\begin{aligned} \bigcup \limits _{k=1}^{N^R} {A_k^S } =\bigcup \limits _{l=1}^{N^T} {T_l }, \quad A_k^S \bigcap \limits _{k\ne i} {A_i^S } =\emptyset . \end{aligned}$$

The task sequence of the *k*-th robot assigned *d* tasks is denoted as $A_k^S=\{a_1^k,a_2^k,\ldots ,a_d^k\}$; each element in $A_k^S$ represents a task $T_l$. Equation (2) ensures that every task is executed only once. In case of problems without cooperative tasks, the time $C_k (A_k ^S)$ of robot $R_k$ finishing all assigned tasks includes the traveling time from the home base of $R_k$ to the first task $a_1^k$, the sum of the traveling time of all pair of assigned tasks $\left( a_i^k \hbox { and } a_{i+1}^k, k\in \{1,2,\ldots ,d-1\}\right)$, the traveling time for returning from the last task $a_d^k$ to the home base of $R_k$, and the sum of the inspection time of all tasks assigned to $R_k$.

**Fig. 6:**
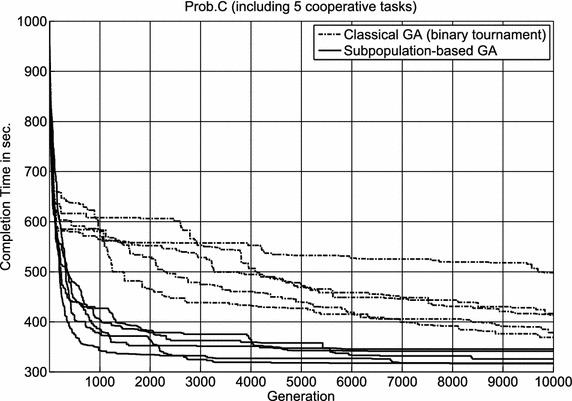
The search progress of two genetic algorithms for solving Prob.C. 5 runs selected from the total 20 runs of each algorithm

For task allocation problems with cooperative tasks, each single-robot task is denoted as a subtask $T_l=t_l$ and each cooperative task (two-robot task) is denoted as two subtasks $T_l=(t_{l1},t_{l2})$, all subtasks would form a set $P=\{P_i \vert i\in \{1,2,\ldots ,N^P\}\}$, i.e.,

3 $$\begin{aligned} \bigcup _{l=1}^{N^T} {T_l }=P. \end{aligned}$$

$N^P=N^T+N^{co}$, $N^{co}$ is the number of cooperative tasks. Instead of $\mathbf A$ in Eq. (1), each admissible solution is denoted as $\mathbf A ^C=\{A_k^C \vert k\in \{1,2,\ldots ,N^R\}\}$ that has to satisfy the following constraint:

4 $$\begin{aligned} \bigcup \limits _{k=1}^{N^R} {A_k^C } =P,\quad A_k^C \bigcap \limits _{k\ne i} {A_i^C } =\emptyset , \end{aligned}$$

with $i,k\in \{1,2,\ldots ,N^R\}$. Where each element in $A_k^C$ represents a subtask $P_i$. Subtasks assigned to the same robot are performed sequentially, while subtasks assigned to different robots are performed in parallel. The constraint (4) ensures that each subtask is executed only once. In case of problems with cooperative tasks, the time $C_k \left( A_k ^C\right)$ of robot $R_k$ finishing all assigned tasks includes the traveling time from the home base of $R_k$ to its first task, the sum of the traveling time between two assigned tasks, the traveling time for returning from the last task to the home base of $R_k$, the sum of the inspection time of all tasks assigned to $R_k$, and the waiting time for carrying out cooperative tasks.

**Fig. 7:**
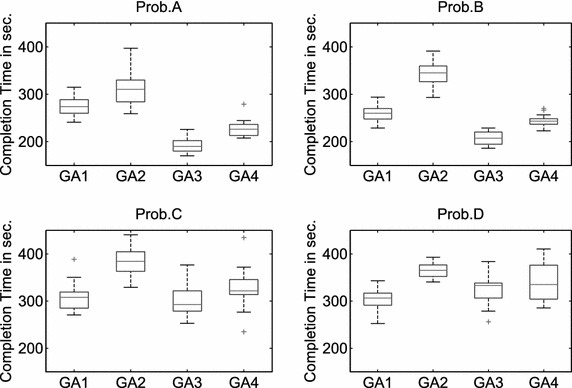
The distribution of the solution quality of the subpopulation-based genetic algorithms with a single mutation operator. 20 runs

In addition, the following three executability constraints (EC) must also be satisfied to ensure that the task allocation $\mathbf A ^C$ of problems with cooperative tasks is feasible for execution:

1. Each cooperative task is carried out by two different robots.
2. Two subtasks of each cooperative task are started at the same time.
3. When two robots are scheduled to carry out a cooperative task, all tasks that are assigned to both robots before this cooperative task have to be executable and finished.

**Fig. 8:**
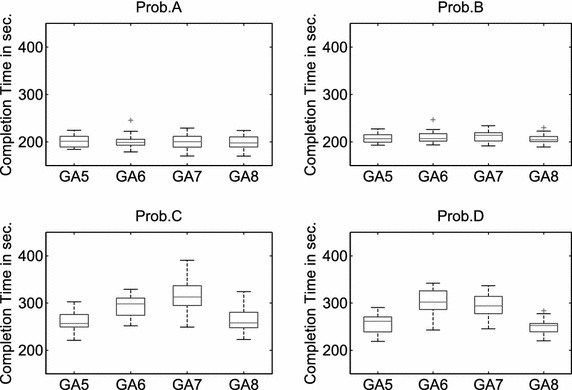
The distribution of the solution quality of the subpopulation-based genetic algorithm with multiple mutation operators. 20 runs

## Methods

A subpopulation-based genetic algorithm is developed to solve the multi-robot task allocation problems with cooperative tasks in this section. At the beginning of this section, the solution representation is introduced. After that, the implementation of the proposed genetic algorithm is illustrated. At the end, the subpopulation-based and standard genetic algorithms (Beyki and Yaghoobi 2015; Larrañaga et al. 1999; Pandey et al. 2016) are compared.

**Fig. 9:**
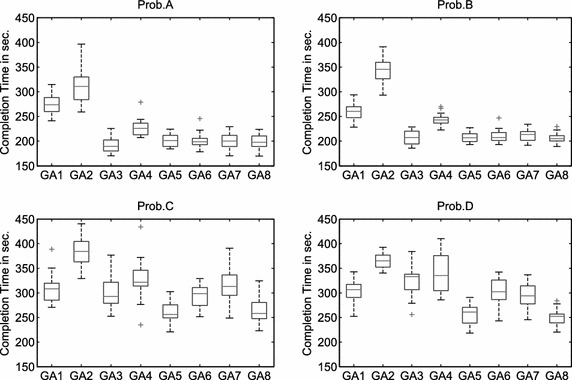
The distribution of the solution quality of the subpopulation-based genetic algorithm with different mutation operators. 20 runs

### Solution representation

Many coding strategies have been proposed to represent solutions, e.g., permutation, adjacency, ordinal coding (Larrañaga et al. 1999). Permutation coding is used to represent a solution in this paper because: (1) it is the most natural and readable way to represent a task sequence; (2) it can be easily implemented in the most commonly used programming languages such as MATLAB or C/C++. Based on the permutation coding, the task-based coding is designed to solve multi-robot task allocation with cooperative tasks. This coding strategy will not create infeasible solutions and a genotype corresponds to just one phenotype. In the following part, the encoding and the decoding of this coding strategy are illustrated, respectively.

**Fig. 10:**
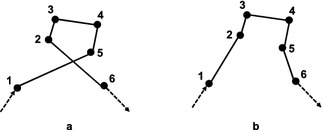
An example with one cross for inversion. **a** Parent. **b** Offspring obtained by inverting {5, 4, 3, 2}

*Encoding (Genotype)* The genotype of a solution represents the task sequence of each robot and consists of two parts:

- A *chromosome* is a string of genes and represents the sequence of all $N^T$ tasks. Each gene represents a task and is denoted as an integer in the range of $[1,N_T]$. Each chromosome consists of $N^T$ distinct integers in the range of $[1,N_T]$.
- A *gene-apportion* is a set of $N^R-1$ distinct integers in the range of $[1,N_T-1]$, which splits a chromosome into $N^R$ parts for $N^R$ robots.

The genotypes represent the initial allocation that each task is assigned to one robot and robot coalitions of cooperative tasks have not yet built. Genetic operators are applied to the genotypes of solutions not phenotypes of them. Both the chromosome and gene-apportion of each genotype in the initial population is generated at random. For example, Fig. 4a shows an example problem where six single-robot tasks ($T_1-T_5,T_8$) and two cooperative tasks ($T_6,T_7$) will be carried out by three robots. In this example, $T_6=(P_6,P_9)$ and $T_7=(P_7,P_8)$, that is, the set of 10 subtasks $P=\{P_1,P_2,P_3,P_4,P_5,P_6,P_7,P_8,P_9,P_{10}\}$. The subtasks and costs are listed in Tables 1, 2, 3, and 4. As each task is encoded as one gene, the genotype of a solution candidate can be represented as shown in Fig. 4c: the chromosome consists of 8 distinct integers in the range of [1, 8], e.g., $\{1,2,3,4,5,6,7,8\}$; this chromosome is split into three segments by a gene-apportion $\{3,6\}$ that is represented as two vertical lines. The phenotype and schedule corresponding to this genotype is shown in Fig. 4d, the strategy of decoding genotypes is detailed in the following parts.

**Table 1:** Coordinates and inspection time of each subtask in Fig. 4

Subtask	$P_1$	$P_2$	$P_3$	$P_4$	$P_5$	$P_6$	$P_7$	$P_8$	$P_9$	$P_{10}$
*x*	3	3	3	7	7	7	7	10	10	10
*y*	13	9	4	3	5	9	13	13	9	4
Inspection time	1	6	1	1	1	1	1	1	1	1

**Table 2:** Coordinates of home base of each robot in Fig. 4

Home base	$S_1$	$S_2$	$S_3$
*x*	4	5	6
*y*	1	1	1

**Table 3:** Traveling time between home bases and subtasks in Fig. 4

Traveling time	$P_1$	$P_2$	$P_3$	$P_4$	$P_5$	$P_6$	$P_7$	$P_8$	$P_9$	$P_{10}$
$S_1$	12.4	8.4	3.4	3.8	5.2	9.2	13.2	16.2	12.2	8.4
$S_2$	12.8	8.8	3.8	2.8	4.8	8.8	12.8	15.8	11.8	7.4
$S_3$	13.2	9.2	4.2	2.4	4.4	8.4	12.4	15.4	11.4	6.4

**Table 4:** Traveling time between any pair of subtasks in Fig. 4

Traveling time	$P_1$	$P_2$	$P_3$	$P_4$	$P_5$	$P_6$	$P_7$	$P_8$	$P_9$	$P_{10}$
$P_1$	0.0	4.0	9.0	11.7	9.7	5.7	4.0	18.7	14.7	13.7
$P_2$	4.0	0.0	5.0	7.7	5.7	4.0	5.7	15.2	11.2	10.2
$P_3$	9.0	5.0	0.0	4.4	4.4	6.7	10.7	14.8	10.8	9.8
$P_4$	11.7	7.7	4.4	0.0	2.0	6.0	10.0	13.0	9.0	6.0
$P_5$	9.7	5.7	4.4	2.0	0.0	4.0	8.0	11.0	7.0	6.0
$P_6$	5.7	4.0	6.7	6.0	4.0	0.0	4.0	13.0	9.0	8.0
$P_7$	4.0	5.7	10.7	10.0	8.0	4.0	0.0	17.0	13.0	12.0
$P_8$	18.7	15.2	14.8	13.0	11.0	13.0	17.0	0.0	4.0	9.0
$P_9$	14.7	11.2	10.8	9.0	7.0	9.0	13.0	4.0	0.0	5.0
$P_{10}$	13.7	10.2	9.8	6.0	6.0	8.0	12.0	9.0	5.0	0.0

**Fig. 11:**
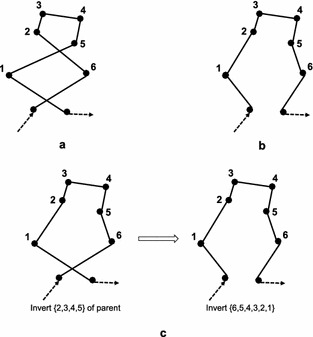
An example with two crosses for swap and inversion. **a** Parent. **b** Offspring obtained by swapping {1} and {6}. **c** Offspring obtained by first inverting {2, 3, 4, 5} and then inverting {6, 5, 4, 3, 2 ,1}

*Decoding (Phenotype)* The phenotypes represent complete feasible task allocations including the distribution and the sequence of all subtasks. The completion time of each solution candidate is calculated according to its phenotype not genotype. In a genotype, each cooperative task is only assigned to one robot, thus the other robot is required to carry out this task simultaneously. A genotype is decoded as a phenotype via two steps:

1. For single-robot tasks, each gene is directly decoded as its corresponding task; see Fig. 5a.
2. For cooperative tasks, two subtasks of each cooperative task should be decoded. The main idea of the following decoding is to minimize the waiting time. According to the genotype, it is obvious that each cooperative task is already assigned to a robot $R_k$, e.g. $T_6$ is assigned to $R_k =R_2$. Hence, the next step is to find the second robot $R_s$ so that two robots can carry it out cooperatively (satisfying the constraint EC1). For each cooperative task $T_l=(P_\alpha ',P_\beta ')$ that is carried out by robot $R_k$ after finishing $P_\gamma$ according to the genotype of an individual, the decoding process is:
  1. One subtask of $T_l =(P_\alpha ',P_\beta ')$, which is closest to robot $R_k$ when it finishing $P_\gamma$, is denoted as $P_\alpha$; the other subtask of $T_l$ is denoted as $P_\beta$. That is, $P_\alpha$ must satisfies $c_{\alpha \gamma k}^t \le c_{\beta \gamma k}^t$, where $c_{ijk}^t$ is the traveling time of robot $R_k$ from one subtask $P_i$ to another subtask $P_j$. Subtask $P_\alpha$ is assigned to robot $R_k$ after finishing $P_\gamma$.
  2. Subtask $P_\beta$ is inserted at the “best” position of the task sequences of robots $R_s\in (R \backslash R_k)$ to satisfy the constraint EC1. Denoting $\tau _i ^a$ as the arriving time of a robot at $P_i$, the waiting time must be $c^w=\left| \tau _\alpha ^a - \tau _\beta ^a \right|$ to satisfy the constraint EC2. If $\tau _\alpha ^a < \tau _\beta ^a$, robot $R_k$ waits for $c^w$ at the inspection position of $P_\alpha$ until robot $R_s$ arrives at the inspection position of $P_\beta$; otherwise robot $R_s$ waits for $c^w$ at the inspection position of $P_\beta$ until robot $R_k$ arrives at the inspection position of $P_\alpha$. The “best” position is the position that provides the least waiting time for performing this cooperative task, which is calculated by enumerating all possible positions of the task sequences of robots $R_s$. To satisfy the constraint EC3, this decoding is carried out starting from the cooperative task that a robot meets first, so that all decoded phenotypes are feasible for execution. The steps of this decoding are:
    (S2.1) Calculate the arriving time $\tau _\alpha ^a$ for all cooperative tasks denoted as a set $T^T$;
    (S2.2) Sort $T^T$ in ascending order by $\tau _\alpha ^a$;
    (S2.3) For the first cooperative task of $T^T$, insert its $P_\beta$ to all possible positions and calculate $c^w$; find the best position that provides the minimal $c^w$; insert $P_\beta$ to the best position, delete this cooperative task from $T^T$, recalculate $\tau _\alpha ^a$.
    (S2.4) Repeat (S2.2)–(S2.3) until all cooperative tasks are decoded.
  For instance, the decoding outcome of the step “S1” is shown in Fig. 5b: $P_6$ is assigned to $R_2$ because $c_{562}^t<c_{592}^t$; $P_7$ is assigned to $R_3$ after leaving its home base (denoted as “0”) because $c_{073}^t<c_{083}^t$. The decoding outcome of the step “S2” is shown in Fig. 5c, d. Possible positions are marked as “active” (denoted as “1”), whereas impossible positions are marked as “inactive” (denoted as “0”). The decoding algorithm first finds the “best” position for $P_9$ because robot $R_2$ meets task $T_6$ earlier than robot $R_3$ meets task $T_7$, i.e., $\tau _6 ^a < \tau _7 ^a$ ($\tau _i^a$ means the arrival time of a robot at the inspection position of $P_i$). There are seven possible positions for decoding $P_9$ except positions of robot $R_2$, but only five positions will be tested: for $R_1$, all four positions are tested; for $R_3$, only the position before $P_7$ is tested because $P_7$ belongs to another cooperative task, which is performed in order to satisfy the constraint EC3. As $P_9$ being inserted before $P_7$ provides the minimum waiting time, $R_2$ is waiting for $c_{62}^w = |\tau _9^a - \tau _6^a| = 0.6$ at $P_6$ until $R_3$ arrives at $P_9$ such that robots $R_2$ and $R_3$ can cooperatively perform $T_6$ (satisfying the constraints EC1 and EC2). In order to satisfy the constraint EC3, positions of chromosomes before either $P_6$ or before $P_9$ are marked as “inactive”; see Fig. 5c. Therefore, only five active positions can be tested when decoding the next cooperative task $P_8$ (see Fig. 5d). Before assigning $P_8$, the arriving time of $R_3$ at $P_7$ is recalculated, $\tau _7^a = 25.4$. The minimum waiting time $c^w=0$ can be obtained when $P_8$ is inserted after $P_6$. That is, $R_2$ arrives at $P_8$ and $R_3$ arrives at $P_7$ at the same time. The complete task allocation obtained using this decoding requires robots $R_2$ and $R_3$ as a coalition to execute $T_6$ and $T_7$ as shown in Fig. 5d. Based on the solution expression in section “Related works on multi-robot task allocation problems”, the solution in Fig. 5 is denoted as $\mathbf A ^C=\left\{ A_1^C,A_2^C,A_3^C\right\} = \{\{P_1,P_2,P_3\}, \{P_4,P_5,P_6,P_8\},\{P_9,P_7,P_{10}\}\}$.

As illustrated above, this decoding can satisfy all executability constraints, i.e., all decoded phenotypes are feasible for execution.

**Fig. 12:**
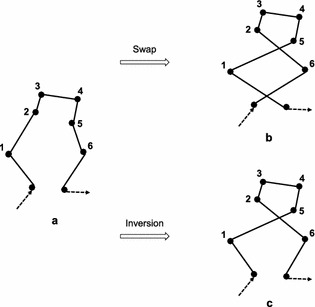
An example of inappropriate swap and inversion. **a** Parent. **b** Offspring obtained by swapping {1} and {6}. **c** Offspring obtained by inverting {2, 3, 4, 5}

Using this representation, each individual (solution candidate) includes a genotype and a phenotype. In the proposed genetic algorithm, the chromosomes of the genotypes are mutated for generating offspring; phenotypes are used to calculate the completion time.

### Process of the developed genetic algorithm

The developed genetic algorithm in this paper is based on subpopulations. The main idea of this algorithm is that selection and genetic operators are applied separately in each subpopulation. Elites selection is based on the fitness of individuals in a subpopulation not in the whole population, thus more elites including both global elites and local elites will be kept in the new generation. These elites avoid losing the best found solution and local optimal solutions. The pseudo code of our proposed subpopulation-based genetic algorithm is presented in Algorithm 1. 
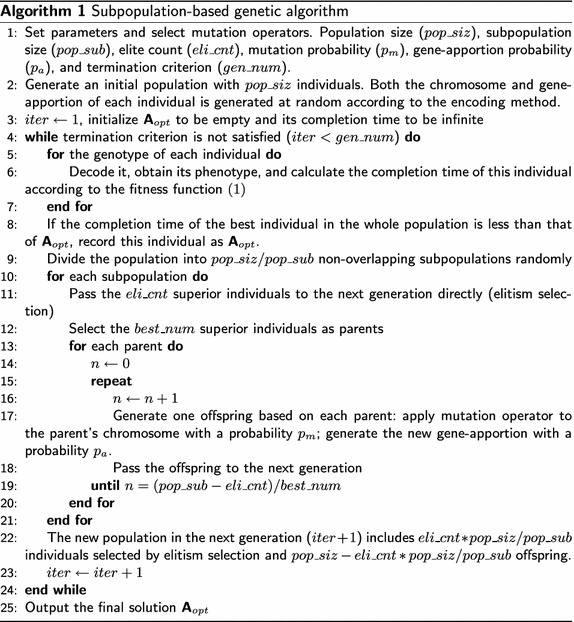


*Parameters* Parameters of the genetic algorithm are set at the beginning, such as population size ($pop\_siz$), subpopulation size ($pop\_sub$), elite count ($eli\_cnt$), mutation probability ($p_m$), and termination criterion ($gen\_num$).

*Initial population* The initial population is randomly produced based on the permutation coding, that is, both the chromosome and gene-apportion of each genotype in the initial population are generated at random based on encoding strategy.

*Fitness calculation* All genotypes should be decoded as phenotypes according to the decoding procedure before fitness calculation. The fitness of each individual means the completion time that is calculated according to the fitness function (1).

*New population* As can be seen from Algorithm 1, a new population is generated based on subpopulations. First, the whole population is randomly divided into non-overlapping subpopulations, and each subpopulation involves $pop\_sub$ individuals. After that, the elitism selection and mutation operators are applied to each subpopulation. The $eli\_cnt$ superior individuals are transferred to the new population, and the $best\_num$ superior individuals are selected as parents. The $pop\_sub-eli\_cnt$ offspring are produced by mutating parents and generating new gene-apportions:

- The chromosome of a new offspring is produced by swap, insertion, inversion, or displacement mutation. *Swap* mutation exchanges two randomly selected genes. *Insertion* mutation moves a randomly chosen gene to another randomly chosen place. *Inversion* mutation reverses a randomly selected gene string. *Displacement* mutation inserts a random string of genes in another random place. Insertion can be considered as a special displacement.
- The gene-apportion of a new offspring is generated with a probability $p_a$; otherwise, the gene-apportion of the parent is kept for the offspring. A gene-apportion is defined by $N^R-1$ integers. Each element in a new gene-apportion is generated by rounding a number that is randomly selected within the range of $[1,N^T]$ according to a standard normal distribution ($\mu , \sigma ^2$). $\mu$ is the cumulative average of the gene-apportion of the best individual obtained in each previous generation; $\sigma =0.03 \times N^T$ is used in this paper. This gene-apportion procedure will choose numbers that are near to the cumulative average, with a higher probability.

*Termination criterion* The genetic algorithm is terminated when the number of generations reaches a predefined number of generations ($gen\_num$) in this paper. Both the population size and the number of generations are fixed in the simulation studies, i.e., the number of all produced individuals is constant. There are many alternative choices of the termination criterion, e.g. a maximal number of generations, CPU time limit, and fitness limit/stall. In this paper, a fixed number of generations is used because (1) CPU time highly depends on the computer hardware, (2) what is a good fitness value is unpredictable, and (3) the convergence properties highly depend on the initial population and the individual run of the genetic algorithm.

### Comparison of the subpopulation-based genetic algorithm and standard genetic algorithms

The main difference between the subpopulation-based genetic algorithm and standard genetic algorithms (Beyki and Yaghoobi 2015; Larrañaga et al. 1999; Pandey et al. 2016) is the way of producing offspring. We compare the subpopulation-based genetic algorithm with standard genetic algorithms with tournament selection (see Algorithm 2) (Beyki and Yaghoobi 2015; Pandey et al. 2016), as the selection strategy of the subpopulation-based genetic algorithm is similar to tournament selection. 
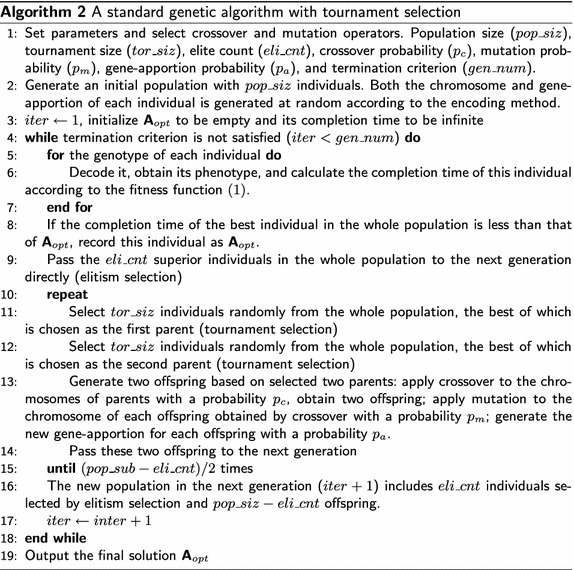


*Selection* First, elites keeping in the new generation are different: a number of superior individuals in the whole population are selected in standard genetic algorithms, while elites in each subpopulation are chosen in the subpopulation-based genetic algorithm. The subpopulation-based genetic algorithm can keep both the current best solution and the local optima that may avoid the population being dominated by a fewer “super” individuals. Second, tournament selection is performed in the whole population randomly, while parents are selected from each non-overlapping subpopulation in the subpopulation-based genetic algorithm. Parents from non-overlapping subpopulations are distributed evenly in the solution space of the current population, which may avoid keeping too many better/worse individuals or missing some local optima.

*Crossover and mutation* Both crossover and mutation are used to produce offspring in standard genetic algorithms, while only mutation operator is used in the proposed genetic algorithm. The $best\_num$ superior individuals in each subpopulation are mutated, while the rest is not used to produce offspring. $pop\_sub-eli\_cnt$ offspring in each subpopulation are generated by mutation with a probability of $p_m=1$. Note that, $p_m=1$ does not mean a random search because: the mutation operator is performed in each subpopulation, and $eli\_cnt$ superior individuals in each subpopulation are kept in the new generation without mutation.

*Time complexity* The procedure of generating a new population in standard genetic algorithms is more complex than that in the proposed subpopulation-based genetic algorithm. The time complexity of the selection in standard genetic algorithms is $O(pop\_siz-eli\_cnt)$, because $pop\_siz-eli\_cnt$ parents are selected. As illustrated above, the time complexity of the selection in the subpopulation-based genetic algorithm is $O(best\_num \cdot pop\_siz/pop\_sub)$. The time complexity of swap is O(1) as it is independent of the chromosome length. The time complexity of insertion, inversion, and displacement is $O(N^T)$ as in the worst case all genes have to be changed. Many crossover alternatives such as partially mapped crossover (PMX) (Goldberg and Lingle 1985), position based crossover, order crossover, and cycle crossover have been proposed for permutation representation. The work of Larrañaga et al. (1999) shows that order crossover is the best crossover and PMX is the fastest crossover when solving small-scale traveling salesman problems. The work of Mudaliar and Modi (2013) shows that PMX is the best crossover when solving traveling salesman problems. The performance of different crossover highly depends on problems. Taking PMX as an example, the mapping relationship between selected *numg* genes from each pair of parents should be built to legalize the offspring. The time complexity of PMX is $O(numg+N^T)$ in the worst case: all *numg* genes should be mapped from one parent to the other and all genes have to be changed.

## Results

In this section, the performance of the proposed genetic algorithm is analyzed when solving multi-robot task allocation problems without/with cooperative tasks. Four problems are tested in the simulation studies:

- *Prob.A* involves 90 single-robot tasks that are distributed in rows; its inspection area is similar to that shown in Fig. 2 but all tasks are single-robot tasks.
- *Prob.B* involves 100 single-robot tasks that are distributed in islands; its inspection area is similar to that shown in Fig. 3 but all tasks are single-robot tasks.
- *Prob.C* involves 80 single-robot tasks and 5 cooperative tasks, and all tasks are distributed in rows; see Fig. 2.
- *Prob.D* involves 90 single-robot tasks and 5 cooperative tasks, and all tasks are distributed in islands; see Fig. 3.

Prob.A and Prob.B are multi-robot task allocation problems without cooperative tasks. Prob.C and Prob.D are multi-robot task allocation problems with cooperative tasks. These scenarios have been used as test cases already in Liu (2014), Liu and Kroll (2015) to compare the performance of different encoding and decoding strategies.

In the experiments, each tested genetic algorithm is performed with a population size of $pop\_siz=200$ and the number of generations chosen as $gen\_num=10^4$. To statistically evaluate the performance of the proposed genetic algorithm, 20 independent runs of each algorithm are implemented on an Intel Core i3 PC with 3.2 GHz, 8 GB (RAM), Windows 7 Professional, MATLAB R2011b. More runs could provide more accurate results but require more CPU time. Hence, 20 independent runs are carried out to restrict the computational effort, and analysis of variance (ANOVA) is used to check whether the performance differences (solution quality) between the different genetic algorithms are statistically significant. If the value of the significance level is smaller than 0.05, the effects of genetic algorithms are assessed to be statistically significant at a level of confidence of 95 %.

### Experiment 1: Subpopulation-based versus binary tournament GA

The first experiment compares the performance of the subpopulation-based genetic algorithm with a standard genetic algorithm with binary tournament selection. The frameworks of both genetic algorithms are displayed in Algorithm 1 and Algorithm 2 in section “Methods”, and the parameters of two genetic algorithms are listed in Table 5. Inversion mutation is used in both genetic algorithms because it performs better than other mutation operators when solving R-permutation problems (Cicirello 2016), such as TSP (Albayrak and Allahverdi 2011; Deep and Mebrahtu 2011; Liu and Kroll 2012b) and flow shop scheduling (Wang and Zhang 2006). As discussed in section “Comparison of the subpopulation-based genetic algorithm and standard genetic algorithms”, a standard genetic algorithm with tournament selection and partially mapped crossover (PMX) is compared with the proposed genetic algorithm because (1) tournament selection is similar to our proposed selection and (2) tournament selection and PMX performs well when solving similar problems (Beyki and Yaghoobi 2015; Mudaliar and Modi 2013; Pandey et al. 2016; Taplin et al. 2005).

**Table 5:** Parameter choice in the experiments

Parameter	Subpopulation-based GA	Standard GA
$pop\_sub$	10	–
$tor\_siz$	–	2
$eli\_cnt$	2	2
$best\_num$	1	–
$p_c$	–	0.9
$p_m$	1	0.01
$p_a$	0.2	0.2
Crossover	–	PMX
Mutation	Inversion	Inversion

**Table 6:** Completion time *J* in sec. and average CPU time in sec. for different genetic algorithms

Problem	Criterion	Subpopulation-based GA	Standard GA
Prob.A	$J_{\mathrm{min}}$	170.06	250.03
	$J_{\mathrm{mean}}$	189.55	290.07
	$J_{\mathrm{max}}$	225.56	319.12
	CPU	988	1432
Prob.B	$J_{\mathrm{min}}$	185.95	257.16
	$J_{\mathrm{mean}}$	207.03	300.45
	$J_{\mathrm{max}}$	228.75	355.11
	CPU	1028	1423
Prob.C	$J_{\mathrm{min}}$	252.72	348.52
	$J_{\mathrm{mean}}$	292.78	414.79
	$J_{\mathrm{max}}$	376.46	500.94
	CPU	2419	2732
Prob.D	$J_{\mathrm{min}}$	255.96	374.93
	$J_{\mathrm{mean}}$	333.07	448.25
	$J_{\mathrm{max}}$	383.95	480.42
	CPU	2580	2885

$J_{\mathrm{max}}$ maximum completion time, $J_{\mathrm{mean}}$ mean completion time, $J_{\mathrm{min}}$ minimum completion time

The experimental results are recorded in Table 6, which indicate that the proposed subpopulation-based genetic algorithm provides better solutions and requires less CPU time than the tested binary tournament genetic algorithm. An ANOVA test shows that the differences in the solution quality between these two genetic algorithms are statistically significant. Randomly choosing 5 from the 20 runs of each genetic algorithm, the solution quality (completion time) of the best solution candidate in each generation is shown in Fig. 6. It is obvious that the subpopulation-based genetic algorithm converges significantly faster than the tested binary tournament genetic algorithm within the first 1000 generations.

This experiment indicates that the proposed genetic algorithm based on subpopulations performs better than the tested binary tournament genetic algorithm with PMX crossover when solving multi-robot task allocation problems, especially when requiring less CPU time and a fewer generations.

### Experiment 2: Subpopulation-based GA with single mutation operator

The second and the third experiments analyze the effects of the subpopulation-based genetic algorithm with different mutation operators and their combinations. Swap, insertion, inversion, and displacement mutation operators are investigated in this paper. The tested subpopulation-based genetic algorithms are listed in Table 7.

**Table 7:** Subpopulation-based genetic algorithm with different mutation operators

Genetic algorithm	Mutation operator(s)
GA1	Swap
GA2	Insertion
GA3	Inversion
GA4	Displacement
GA5	Swap and inversion
GA6	Insertion and inversion
GA7	Displacement and inversion
GA8	Swap, insertion, inversion, and displacement

The second experiment tests the performance of the subpopulation-based genetic algorithms with a single mutation operator (GA1–GA4 in Table 7); each mutation operator produces $pop\_sub-eli\_cnt=8$ offspring in each subpopulation. The results are shown in Fig. 7. An ANOVA test shows that: (1) inversion (GA3) performs significantly better than the other three mutation operators when solving Prob.A and Prob.B; (2) the differences in the solution quality are not statistically significant when using swap, inversion, and displacement to solve Prob.C and Prob.D.

### Experiment 3: Subpopulation-based GA with multiple mutation operators

The third experiment analyzes the performance of the subpopulation-based genetic algorithms with multiple mutation operators (GA5–GA8 in Table 7). Each mutation operator in GA5–GA7 produces 4 offspring in each subpopulation by repeated application; each mutation operator in GA8 produces 2 offspring in each subpopulation; all mutation operators are applied in parallel. Inversion is combined with the other mutation operators in this experiment, because it performed well in the second experiment. The experimental results are displayed in Fig. 8. An ANOVA test shows that: (1) the differences in the solution quality between GA5–GA8 are not statistically significant when solving Prob.A and Prob.B; (2) GA5 and GA8 can provide significantly better solutions than GA6 and GA7 when solving Prob.C and Prob.D.

**Table 8:** Completion time *J* in sec. for the subpopulation-based genetic algorithm with different mutation operators

Problem	Criterion	GA1	GA2	GA3	GA4	GA5	GA6	GA7	GA8
Prob.A	$J_{\mathrm{min}}$	240.87	259.00	170.06	207.28	184.07	178.41	170.10	*169.73*
	$J_{\mathrm{mean}}$	273.98	310.50	*189.55*	225.93	200.86	198.31	200.00	197.73
	$J_{\mathrm{max}}$	314.81	396.67	225.56	278.89	224.40	245.39	229.00	*223.99*
Prob.B	$J_{\mathrm{min}}$	228.42	293.00	*185.95*	222.65	193.22	193.25	191.36	189.00
	$J_{\mathrm{mean}}$	260.16	345.23	207.03	243.05	206.74	206.91	213.62	*204.46*
	$J_{\mathrm{max}}$	294.05	390.84	228.75	270.26	*227.21*	246.90	234.23	229.72
Prob.C	$J_{\mathrm{min}}$	270.50	328.82	252.72	234.96	*220.76*	251.82	248.93	222.82
	$J_{\mathrm{mean}}$	308.14	384.37	292.78	321.59	*256.44*	298.16	312.96	257.80
	$J_{\mathrm{max}}$	388.62	440.42	376.46	434.31	*302.74*	328.96	390.78	324.31
Prob.D	$J_{\mathrm{min}}$	252.29	340.35	255.96	285.73	*218.58*	242.96	245.62	220.00
	$J_{\mathrm{mean}}$	306.57	365.23	333.07	335.02	261.11	302.09	294.19	*252.44*
	$J_{\mathrm{max}}$	343.06	392.85	383.95	410.17	290.83	342.09	336.63	*283.71*

Best results highlighted in italic face

GA1—Swap; GA2—Insertion; GA3—Inversion; GA4—Displacement; GA5—Swap and inversion; GA6—Insertion and inversion; GA7—Displacement and inversion; GA8—Swap, insertion, inversion, and displacement. ${}^{2}$ Prob.A and Prob.B without cooperative tasks; Prob.C and Prob.D with cooperative tasks

The results of all tested subpopulation-based genetic algorithms listed in Table 7 are shown in Fig. 9 and Table 8. GA3, GA5, and GA8 can provide better solutions than the other genetic algorithms. An ANOVA test shows that: (1) the differences in the solution quality using GA3, GA5, GA6, GA7, and GA8 are not statistically significant when solving Prob.A and Prob.B; (2) GA5 and GA8 perform significantly better than the other tested genetic algorithms when solving Prob.C and Prob.D. 

The implementation of the subpopulation-based genetic algorithm and test results for solving Prob.C is available as Additional file 1.

## Discussion

Experimental results show that inversion performs well when solving multi-robot task allocation problems without cooperative tasks, which is similar to the study of solving traveling salesman problems (Albayrak and Allahverdi 2011; Deep and Mebrahtu 2011; Liu and Kroll 2012b). The swap and inversion combination performs well when solving multi-robot task allocation problems with cooperative tasks, which could be due to the fact that they can improve solution candidates with crossed paths effectively.

In general, it is difficult to find the *best* mutation operator that could produce all desired effects. The influences of mutation operators vary in different genetic algorithms and in solving different problems. According to what most influences solution fitness, permutation problems were be classified into three major types (Cicirello 2015, 2016; Cicirello and Cernera 2013; Sörensen 2007). Cicirello (2016) theoretically analyzed the performance of several common mutation operators on different permutation problems, and suggested swap for most A-permutation problems and inversion for R-permutation problems with undirected edges.

Multi-robot task allocation problems studied in this paper are blended-permutation problems, as both task assignments among robots and task scheduling for each robot impact the fitness. Therefore, the performance of mutation operators should be analyzed based on specific permutation problems.

In industrial plant inspection problems, good task allocations usually do not include crossed paths or only include a few crossed paths. Figure 10a shows an example where one cross may occur. Transforming permutation $\{1,5,4,3,2,6\}$ (Fig. 10a) into $\{1,2,3,4,5,6\}$ (Fig. 10b) needs at least: one inversion, that is, inverting $\{5,4,3,2\}$; or two swaps, that is, swaping $\{5\}$ and $\{2\}$, then swaping $\{4\}$ and $\{3\}$; or three insertions/displacements, that is, inserting $\{2\}$, $\{3\}$, $\{4\}$ before $\{5\}$ sequentially. Figure 11a shows another example where two crosses may occur. Transforming permutation (a) to (b) in Fig. 11 needs at least: one swap, that is, swapping $\{1\}$ and $\{6\}$, see Fig. 11b; two inversions/insertions/displacements, see Fig. 11c, d. These two examples imply that proper swap is more efficient than inversion in case of many crossed paths. On the contrary, inappropriate swap produces worse solutions than inversion, e.g. swap produces two crosses, while inversion produces one cross in Fig. 12. Therefore, inversion can obtain better results than swap if given a large number of generations.

For mutation combinations, it is difficult to analyze which is the only operator that guides the evolutionary search, because: (1) an operator cannot guarantee to produce better offspring than parents; and (2) the best individual in $n+1$ generation may not be generated by mating the best individual in *n* generation. Although there are no significant differences between results of GA5 and GA8, it cannot indicate that insertion and displacement mutation operators have no effect on the evolutionary search. Therefore, it cannot be said that one specific mutation operator is *the best*. Based on the experimental results, multiple mutation operators (including both inversion and swap) is suggested when solving similar combinatorial optimization problems.

## Conclusion

The problem complexity significantly increases if cooperative tasks are involved because they introduce additional spatial and temporal constraints. To solve the multi-robot task allocation problems without/with cooperative tasks for industrial plant inspection, a subpopulation-based genetic algorithm is developed. The proposed subpopulation-based genetic algorithm using just inversion mutation and selection obtains better solutions than the tested binary tournament genetic algorithm with partially mapped crossover (PMX) and inversion mutation. This provides the possibility of crossover-free genetic algorithms. Succeeding, the impact of four mutation operators and four mutation operator combinations in the subpopulation-based genetic algorithm is analyzed to find suitable mutation operators for multi-robot task allocation problems. The results indicate that inversion mutation performs well when solving problems without cooperative tasks, and the swap-inversion combination performs well when solving problems with cooperative tasks. As it is difficult to produce all desired effects with a single mutation operator, using multiple mutation operators (including both inversion and swap) is suggested when solving similar combinatorial optimization problems.

## Additional file

10.1186/s40064-016-3027-2 The implementation of the subpopulation-based genetic algorithm and test results for solving Prob.C can be downloaded from https://figshare.com/s/752db35c921eea01f988.
