# Supplementary material for: Performance impact of mutation operators of a subpopulation-based genetic algorithm for multi-robot task allocation problems
Source: Springerplus. 2016 Aug 18;5(1):1361. doi: 10.1186/s40064-016-3027-2 (PMC4990531; doi:10.1186/s40064-016-3027-2)
Supplement: Supplementary file 1 — 10.1186/s40064-016-3027-2 The implementation of the subpopulation-based genetic algorithm and test results for solving Prob.C can be downloaded from https://figshare.com/s/752db35c921eea01f988. [file 40064_2016_3027_MOESM1_ESM.zip › Prob.C/Readme.pdf]

Introduction to Source Code and Test Data

Chun Liu
School of Automation, Beijing University of Posts and Telecommunications, China
Department of Measurement and Control, University of Kassel, Germany
chun.liu@bupt.edu.cn

There are total 4 issues in this zip file, including 3 folders and 1 PDF file, i.e.,
(1) Initial Population
(2) Results
(3) Source Code
(4) Readme
The above documents is used for solving Prob.C, which involves 80 single-robot tasks and 5 cooperative tasks. The code can be used to solve other problems in the manuscript.

1. Initial Population

20 initial populations for testing 20 runs/each genetic algorithm

2. Results

Because some of the result files are too large, only the results of solving Prob.C are provided.

Result files	GA	Mutation operator
Case1PA_CO_LW_SW_TS10A_2_pop200_run	GA1	Swap
Case1PA_CO_LW_IS_TS10A_2_pop200_run	GA2	Insertion
Case1PA_CO_LW_IV_TS10A_2_pop200_run	GA3	Inversion
Case1PA_CO_LW_DP_TS10A_2_pop200_run	GA4	Displacement
Case1PA_CO_LW_IVIS_TS10A_2_pop200_run	GA5	Swap and inversion
Case1PA_CO_LW_IVSW_TS10A_2_pop200_run	GA6	Insertion and inversion
Case1PA_CO_LW_IVDP_TS10A_2_pop200_run	GA7	Displacement and inversion
Case1PA_CO_LW_ALL_TS10A_2_pop200_run	GA8	Swap, insertion, inversion, and displacement

3. Source Code of GA

GA is implemented with MATLAB2010 and higher.
1) **.mat files**: data required in GA.

Files name	Variants	Explanation
Astarcost1	distanrt	Traveling cost from robot to task (for GA fitness calculation)
	distantt	Traveling cost from task to task (for GA fitness calculation)
	crt	Traveling path from robot to task (for show figure)
	ctt	Traveling path from task to task (for show figure)
	cluster	is not used in this test (used for different coding strategies, which are proposed in our previous work cited in the paper)
EncodingLW		coding strategy used in this paper, the relationship between tasks and subtasks
map2013	MAP	grid map as matrix (for show figure)

	MAX_X,MAX_Y	boundary of coordinates
	robot	coordinates of robots(for show figure)
	task	coordinates of inspection positions, the inspection time at each position, and the id of cooperative tasks
	xarea	inadmissible areas

2) **.m files**: MATLAB script and function

Files name	Explanation
fitfunction_2013TBLW	fitness function - costs of each individual
GA_2013TBLW_SW_TS10APRO	subpopulation-based genetic algorithm with swap mutation
GA_2013TBLW_IS_TS10APRO	subpopulation-based genetic algorithm with insertion mutation
GA_2013TBLW_ IV_TS10APRO	subpopulation-based genetic algorithm with inversion mutation
GA_2013TBLW_DP_TS10APRO	subpopulation-based genetic algorithm with displacement mutation
GA_2013TBLW_IVSW_TS10APRO	subpopulation-based genetic algorithm with inversion and swap mutation
GA_2013TBLW_IVIS_TS10APRO	subpopulation-based genetic algorithm with inversion and insertion mutation
GA_2013TBLW_IVDP_TS10APRO	subpopulation-based genetic algorithm with inversion and displacement mutation
GA_2013TBLW_ALL_TS10APRO	subpopulation-based genetic algorithm with swap, insertion, inversion and displacement mutation
main	main function
mapget2013	show map of inspection problem
randbreaksdiss	generate gene-apportion
show_solution	show the final solution

3) **.fig file**: newmap.fig displays the map of inspection problem.
